# Structural correlates of survival in Progressive Supranuclear Palsy

**DOI:** 10.1016/j.parkreldis.2023.105866

**Published:** 2023-10-02

**Authors:** Duncan Street, W Richard Bevan-Jones, Maura Malpetti, P Simon Jones, Luca Passamonti, Boyd CP Ghosh, Timothy Rittman, Ian TS Coyle-Gilchrist, Kieren Allinson, Catherine E Dawson, James B Rowe

**Affiliations:** 1 Department of Clinical Neurosciences and Cambridge University Hospitals NHS Trust, University of Cambridge, UK; 2 Department of Psychiatry, University of Cambridge, UK; 3 Consiglio Nazionale delle Ricerche (CNR), Istituto di Bioimmagini e Fisiologia Molecolare (IBFM), Milano, Italy; 4 Wessex Neurological Centre, University Hospitals Southampton NHS Foundation Trust, Southampton, UK; 5 Norfolk and Norwich NHS Foundation Trust, Norwich, UK; 6 Department of Pathology, Cambridge University Hospitals NHS Trust, Cambridge, UK; 7 MRC Cognition and Brain Sciences Unit, University of Cambridge, Cambridge, UK

## Abstract

**Introduction:**

Many studies of the Richardson’s syndrome phenotype of progressive supranuclear palsy (PSP) have elucidated regions of progressive atrophy and neural correlates of clinical severity. However, the neural correlates of survival and how these differ according to variant phenotypes are poorly understood. We set out to identify structural changes that predict severity and survival from scanning date to death.

**Methods:**

Structural magnetic resonance imaging data from 112 deceased people with clinically defined ‘probable’ or ‘possible’ PSP were analysed. Neuroanatomical regions of interest volumes, thickness and area were correlated with ‘temporal stage’, defined as the ratio of time from symptom onset to death, time from scan to death (‘survival from scan’), and in a subset of patients, clinical severity, adjusting for age and total intracranial volume. Forty-nine participants had *post mortem* confirmation of the diagnosis.

**Results:**

Using T1-weighted magnetic resonance imaging, we confirmed the midbrain, and bilateral cortical structural correlates of contemporary disease severity. Atrophy of the striatum, cerebellum and frontotemporal cortex correlate with temporal stage and survival from scan, even after adjusting for severity. Subcortical structure-survival relationships were stronger in Richardson’s syndrome than variant phenotypes.

**Conclusions:**

Although the duration of PSP varies widely between people, an individual’s progress from disease onset to death (their temporal stage) reflects atrophy in striatal, cerebellar and frontotemporal cortical regions. Our findings suggest magnetic resonance imaging may contribute to prognostication and stratification of patients with heterogenous clinical trajectories and clarify the processes that confer mortality risk in PSP.

## Introduction

Survival in the Richardson’s syndrome phenotype of progressive supranuclear palsy (PSP) is typically 5 to 7 years from symptom onset.^1–6^ Midbrain and frontal lobe atrophy are characteristically associated with the presence of PSP^7–13^ and imaging changes can differentiate Richardson’s syndrome from other ‘parkinsonian’ syndromes as well as correlating with clinical features and severity.^10,14–18^ There is also extensive evidence from imaging^14,15,19–23^ and *post mortem* studies^24,25^ for the importance of white matter pathology in PSP. Progressive atrophy has been demonstrated and linked to disease progression and severity of clinical features.^26,27^

**Figure 1:**
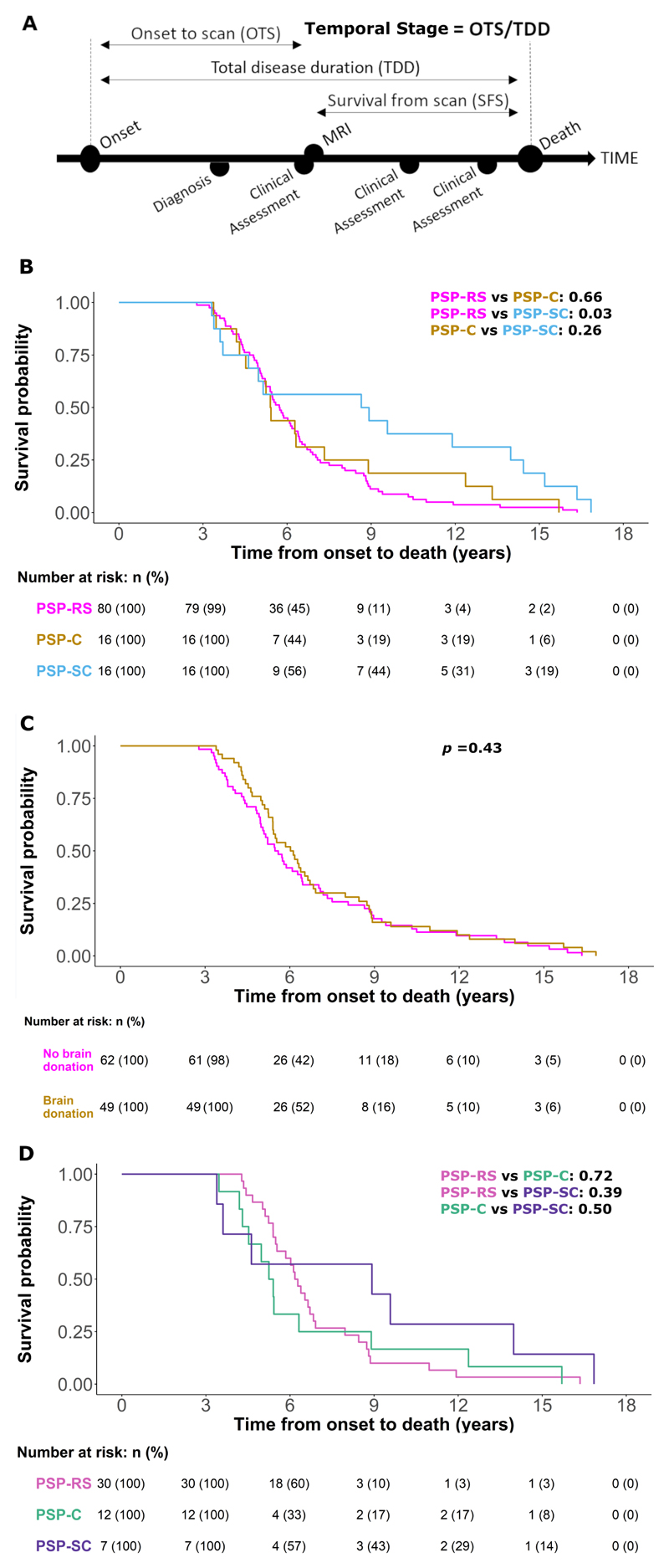
(A): Study schematic and definitions of key variables. Temporal stage (‘stage’) is defined as the time from symptom onset to MRI (time to MRI, TTM) divided by the time from symptom onset to death (total disease duration, TDD) (B, C & D): Survival analysis from symptom onset to death. Analysis was performed using a Cox regression model split according to diagnostic groups with associated number at risk tables below each plot. Displayed p-values represent pairwise log-rank comparisons with correction for multiple comparisons. (B) PSP-Richardson’s syndrome (PSP-RS), PSP-Cortical (PSP-C) and PSP-Subcortical (PSP-SC) groups. (C) Groups split according to those who donated their brain to the Cambridge Brain Bank (‘Brain donation’) and those who did not (‘No brain donation’). PSP = progressive supranuclear palsy, MRI = magnetic resonance imaging. (D) PSP-Richardson’s syndrome (PSP-RS), PSP-Cortical (PSP-C) and PSP-Subcortical (PSP-SC) groups in the pathologically confirmed subset

Whilst several clinical features have been correlated with survival,^2,6,28–31^ the neuroanatomical predictors or determinants of survival time after a scan remain poorly understood. As proposed in the Neuroimaging Biomarker Utility System for PSP,^32^ the correlates of survival may differ from the imaging hallmarks of PSP (‘diagnosis’), or the correlates of motor and cognitive severity (‘severity’), or those regions with progressive atrophy (‘progression’). If, for example, clinical feature A is particularly associated with higher risk of death (e.g., bulbar failure), but clinical features B and C contribute strongly to a clinical severity rating scale (e.g., limb and ocular signs), then the anatomical correlates of survival (mediated by A) would differ from the anatomical correlates of severity (represented by B and C). Individual clinical measures may also vary in their dynamic range, with floor or ceiling effects that obscure progression of disease. The survival following an assessment is usually considered in terms of absolute time, for example, months between diagnosis/symptom onset and death. However, survival time varies greatly between people with PSP. To compare across individuals, progression through the illness can also be considered as a proportion of the duration of the disease to its full course, providing a normalised measure of survival. We will refer to this proportion of time from onset to end of illness as the ‘temporal stage’ (see Figure 1, panel A). This ‘temporal stage’ differs from a clinical severity-based staging of PSP, or the identification of progressive atrophy, which underlie many former studies. Improved understanding of the mechanisms underlying progression and survival, and predicting them, is important for stratification in clinical trials design, and for targeting factors that mediate survival.

In recent years, a focus of phenotypic variation within PSP neuroimaging has been the identification of brainstem derived biomarkers. Compared to Richardson’s syndrome, PSP-parkinsonism (PSP-P) has been found to have a greater midbrain area,^33–35^ decreased pons to midbrain area ratio,^33,35–37^ increased superior cerebellar peduncle width^33,34,38^ and decreased magnetic resonance parkinsonism index score.^37,39,40^ Similarly, midbrain area and pons to midbrain area ratio have been found to differ between Richardson’s syndrome and PSP-progressive gait freezing (PSP-PGF),^35,41^ PSP- speech/language (PSP-SL),^41^ PSP-corticobasal syndrome (PSP-CBS)^35^ and grouped variant diagnoses.^42^ When considering non-brainstem markers, frontal cortical atrophy was more marked in PSP-CBS, PSP-frontal (PSP-F) and PSP-SL variants than Richardson’s syndrome, while dentatorubrothalamic tract involvement was only observed in PSP-CBS, PSP-F and Richardson’s syndrome in the largest study of its type using 2017 Movement Disorders Society (MDS) diagnostic criteria.^43^

It is unknown how structural variation predicts prognosis and survival. Our primary hypothesis was that structural change (atrophy) predicts survival, at least in terms of the ‘temporal stage’ from onset to death when the scan was undertaken. Our second hypothesis was that although there are structural correlates of disease severity (e.g., PSP-Rating Scale, PSPRS), they differ from those associated with survival. Our third hypothesis was that neuroanatomical correlates of severity and survival differ by disease phenotype. Specifically, we predicted stronger subcortical regional correlations in PSP-Subcortical phenotypes (i.e., PSP-P and PSP-PGF) than PSP-Cortical phenotypes (i.e., PSP-CBS, PSP-SL and PSP-F).

## Methods

### Participants

Between June 2007 and June 2019, 122 people with a contemporary clinical diagnosis of probable or possible PSP underwent standardised structural imaging. They were retrospectively classified according to 2017 MDS criteria for a diagnosis of ‘probable’ or ‘possible’ PSP.^44^ Cases of multiple PSP diagnoses at presentation were resolved under the hierarchical multiple allocations extinction criteria (MAX rules).^45^ All participants had died by the census date of 31^st^ August 2020 and neuropathological confirmation was available for 49 patients who donated their brain to the Cambridge Brain Bank. ‘Onset to scan’ (OTS) was defined as time from the first recognised symptom onset to MRI scan, ‘total disease duration’ (TDD) defined as time from the first symptom onset to death and ‘survival from scan’ (SFS) defined as time from MRI scan to death. The ‘temporal stage’ was defined as the fraction OTS/TDD, for each participant (Figure 1, panel A).

The study was conducted in accordance with the 1964 Helsinki declaration. Participants gave written, informed consent. Ethical approval was granted in October 2015 for the ‘Pick’s Disease and Progressive Supranuclear Palsy Prevalence and Incidence’ protocol (12/EE/0475) by the East of England Cambridge Central Research Ethics Committee and in March 2007 for the ‘Diagnosis and prognosis in Progressive Supranuclear Palsy and Corticobasal Degeneration’ protocol (07/Q0102/3) by the East of England Essex Research Ethics Committee. Neuropathological data were obtained with ethical approval from the Health Research Authority, NHS England (IRAS—202 802, ‘Neurodegeneration Research in Dementia’).

### Acquisition of MRI

Participants underwent T1-weighted magnetic resonance imaging between 2007 and 2019 at the Wolfson Brain Imaging Centre, University of Cambridge, UK, on various Siemens scanners, all with 3T field strength. Visual inspection was performed to identify and label participants with large ventricles and ten individuals were excluded on grounds of image quality or lesion, leaving 86 TrioTim, 15 PRISMA, 6 Skyra and 5 Verio scans. The T1-weighted magnetization-prepared rapid acquisition gradient-echo sequence (MPRAGE) was closely matched across scanners based on the international Genetic Frontotemporal Dementia Initiative protocols (repetition times 2000ms, echo time 2.93ms, Flip angle 8°, 1.1mm isotropic).^46^ Where participants had more than one scan during the study period, the scan chosen for analysis was selected according to (i) the highest image quality, with least motion artefact, and (ii) the shortest interval between scan and PSPRS evaluation.

### The Volumetric Analysis

Pre-processing of the MPRAGE images used automated scripts in the Statistical Parametric Mapping software (SPM12) (www.fil.ion.ucl.ac.uk/spm/software/spm12) in Matlab 2019b (mathworks.com). Cortical reconstruction and volumetric parcellation were performed using the standard recon-all pipeline of FreeSurfer 6.0.0 (Massachusetts General Hospital, Harvard Medical School; http://surfer.nmr.mgh.harvard.edu/) with adjustments for large ventricles where appropriate, and additional brainstem structures parcellation. Regional analyses were performed using cortical grey matter volume measures from 68 Desikan-Killiany Atlas cortical regions and 38 subcortical volume measures from the segmentation were applied.^47,48^ Total intracranial volume (TIV) was calculated separately using Statistical Parametric Mapping (SPM12).

Output from the Freesurfer pipeline was then imported to R Studio (version 4.0.3) to determine group differences and assess the relationship of outcome measures with regional brain parameter. Regional parameters of interest were cortical thickness, cortical area, cortical volume, and subcortical volume. Generalised linear mixed models were constructed including temporal stage, phenotype (e.g., Richardson’s syndrome, PSP-Cortical and PSP-Subcortical) and survival from scan separately as covariates of interest, while age, TIV and PSPRS were included as covariates of no interest across all models. Sex differences were subsumed under the TIV covariate. Pearson’s partial correlations were used to explore relationships between PSPRS, temporal stage and survival from scan. Correlation coefficients were converted using Fisher’s *r* to *z* transformation and compared via calculation of the observed *z* test statistic. Following these analyses, we constructed a simple exploratory multiple regression model constituting the top 5 imaging region-of-interest parameters and baseline characteristics to estimate the predictive accuracy of consequent survival (i.e., the difference between actual date of death and the date predicted by the model in key brain regions). Regional z-values were calculated for each patient using region-specific mean and standard deviation. For all regional analyses, significance levels were assessed using false discovery rate (FDR) correction. An FDR adjusted *p*-value <0.05 was considered significant.

## Results

### Clinical results

Clinical and demographic features are summarised in Table 1A. A Kaplan-Meier survival curve for the cohort by clinical diagnosis (median survival 5.73 years) is illustrated in Figure 1, panel B. As expected, ‘temporal stage’ and ‘survival from scan’ were significantly correlated (Pearson r = -0.689, p<10^-8^). The PSPRS was significantly correlated with ‘temporal stage’ (Pearson r = 0.538, p<10^-6^) and ‘survival from scan’ (Pearson r = -0.319, p = 0.004). The Subcortical group were diagnosed over two years later on average after symptom onset than the Richardson’s syndrome group whereas the Cortical group were diagnosed after a similar interval.

### Neuropathology

Neuropathology was examined in forty-nine participants in the study. Forty-six (46/49 = 93.9%) had typical PSP pathology. Two (2/49 = 4.1%) had tau pathology that was intermediate between PSP and corticobasal degeneration, in anatomical distribution and neuroglial involvement (but lacking ballooned achromatic neurons). One case had dual pathology with the combination of typical PSP tauopathy plus cortical Lewy bodies. Demographic and clinical information for this sub-group is provided in Table 1B. The Kaplan-Meier survival curve for the 49 participants who donated to the brain bank was not different from that who did not donate (Log rank 0.43) (Figure 1, panel C). No significant differences in survival probability according to phenotype were observed in the pathologically confirmed group (Figure 1, panel D).

### Imaging results

TrioTim scans constituted the majority in each phenotypic group: 63/80 (78.8%) in Richardson’s syndrome, 12/16 (75.0%) in the Cortical group and 11/16 (68.8%) in the Subcortical group. The same pattern was observed in the pathologically confirmed subset: 24/30 (80.0%) in Richardson’s syndrome, 10/12 (83.3%) in the Cortical group and 5/7 (71.4%) in the Subcortical group. Across all analyses no significant differences were observed between scanner type, in age, sex, clinical severity (as measured by PSP Rating Scale) or phenotypic group.

**Figure 2:**
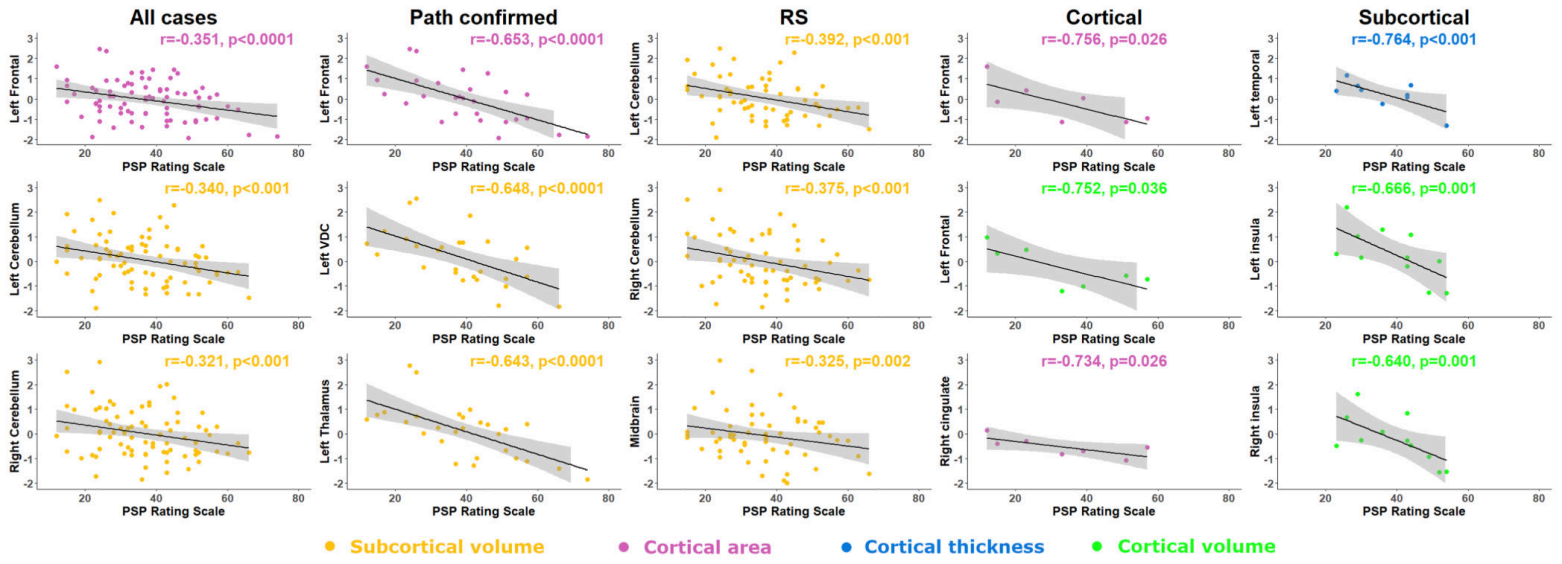
Structural correlates of severity. Pearson’s r partial correlation coefficient analysis between Progressive Supranuclear Palsy Rating Scale and region-of-interest z-values according to disease phenotype. RS – Richardson’s syndrome.

Results from partial correlation analyses between regional parameters and total PSPRS score are displayed in Figure 2, Table 2, Supplementary Figure 1, and Supplementary Tables 1, 4 and 5. Midbrain volume correlated with PSPRS in all participants (r= -0.286, n=80, p=0.002) and in the Richardson’s syndrome group (r= -0.325, n= 62, p=0.002), with a similar but stronger relationship observed in the pathologically confirmed group (r= -0.587, n=28, p<0.001). Pons volume also correlated with PSPRS in all participants (r= -0.194, n=80, p=0.039), the pathologically confirmed group (r= -0.451, n=28, p=0.001) and the Richardson’s syndrome group (r= -0.221, n=62, p=0.044). Outside the brainstem, broadly symmetrical patterns of volumetric correlation with PSPRS were observed in the ventral diencephalon, cerebellum, frontal, temporal, occipital and cingulate cortex of the whole group (combined phenotypes), and the ventral diencephalon and cerebellum of the Richardson’s syndrome group. Left frontal cortical area showed strong correlation with PSPRS in the whole group (r = -0.351, n=80, p<0.0001) and the pathologically confirmed group (r = -0.653, n=28, p<0.0001). Left temporal thickness showed strong correlation in the Subcortical group (r = -0.764, n=11, p<0.001) and frontal, temporal and cingulate volumes also showed broadly symmetrical correlation with PSPRS in the Subcortical group, and these differed from the Richardson’s syndrome group. Left frontal cortical area (r = -0.756, n=7, p=0.026) and right cingulate cortical area (r = -0.734, n=7, p=0.026) showed strong correlation with PSPRS in the Cortical group but no significant correlations were observed between subcortical regional volumes and PSPRS in the separate Cortical or Subcortical subgroups analyses.

**Figure 3:**
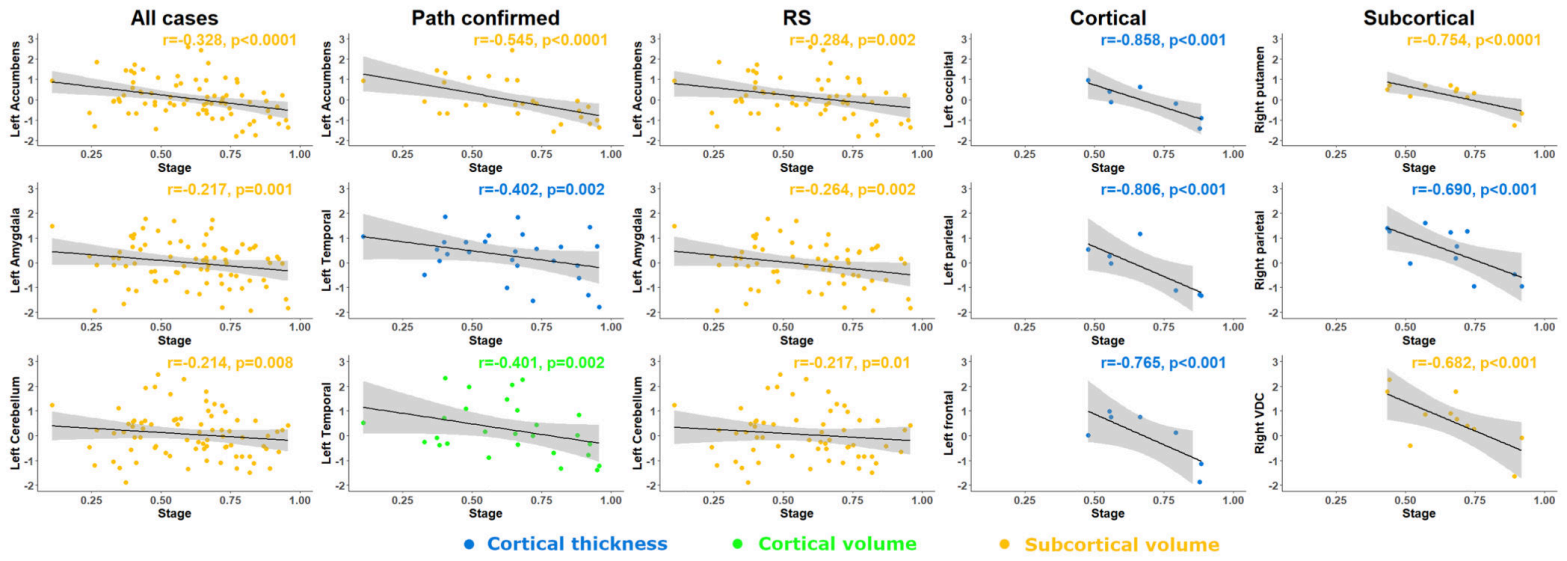
Structural correlates of survival in terms of ‘temporal stage’ (ratio from onset to death). Pearson’s r partial correlation coefficient analysis between ‘temporal stage’ and region of interest z-values according to disease phenotype. RS – Richardson’s syndrome.

Regional partial correlation analyses with ‘temporal stage’ are displayed in Figure 3, Table 2, Supplementary Figure 2 and 3, and Supplementary Tables 6 and 7. The putamen, accumbens, ventral diencephalon, frontal and temporal cortex volumes showed strong correlation bilaterally with proximity to the end of the illness (i.e., temporal stage) in both the Subcortical and pathologically confirmed groups. The putamen and cerebellum showed similar but weaker negative correlations with more advanced temporal stage in the Richardson’s syndrome group. The Cortical and Subcortical groups showed strong bilateral negative correlations with multi-region cortical thickness and temporal stage but did not display any symmetrical volumetric correlates with temporal stage. No significant symmetrical correlations between cortical area and temporal stage were observed in any group. Inter group differences in correlation coefficients of temporal stage were most marked between Richardson’s syndrome and Subcortical groups. No differences in correlation coefficients of regional volumes and temporal stage were observed between Richardson’s syndrome and Cortical groups.

**Figure 4:**
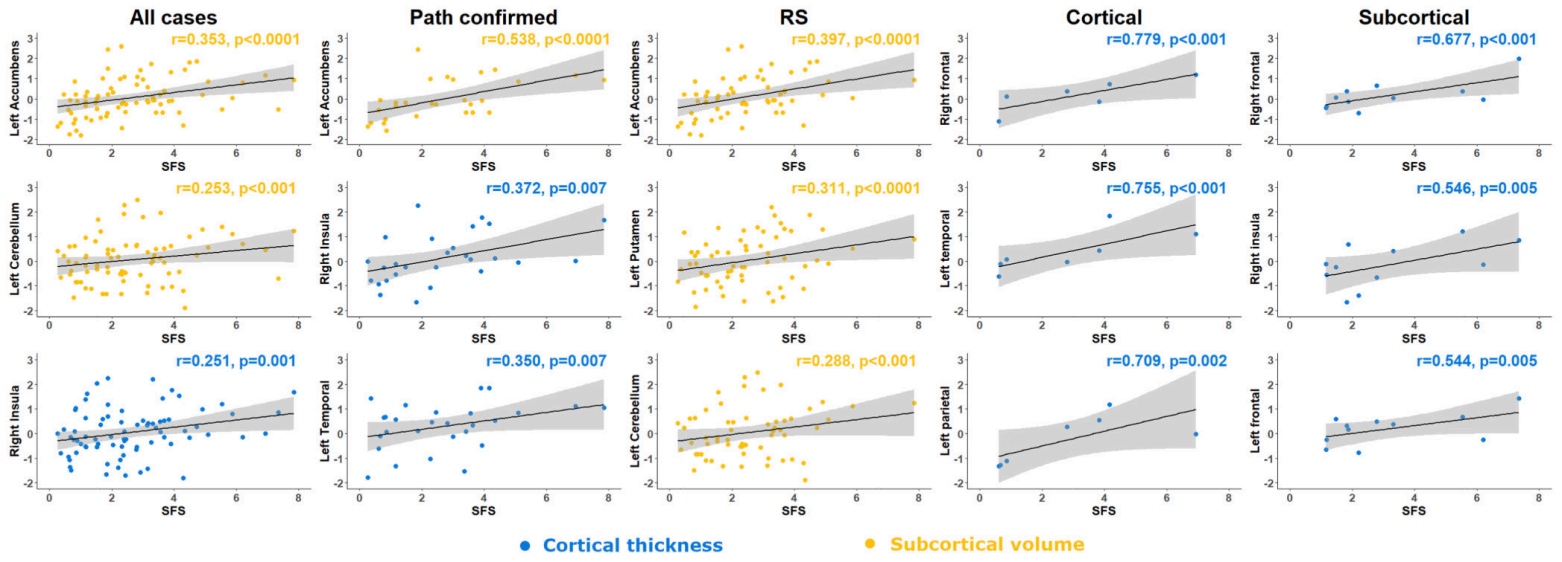
Structural correlates of survival in terms of survival from scan. Pearson’s r partial correlation coefficient analysis between survival from scan and region of interest z-values according to disease phenotype. RS – Richardson’s syndrome.

Regional partial correlation analyses with ‘survival from scan’ are displayed in Figure 4, Table 2, Supplementary Figures 2 and 3 and Supplementary Tables 3, 8 and 9. The putamen, hippocampus and accumbens regional volumes showed strong negative correlation bilaterally with shorter survival from scan in all participants, while the pallidum and accumbens also negatively correlated symmetrically with shorter survival from scan in the Richardson’s syndrome group. In other words, more atrophy was associated with shorter survival, *even after adjusting for clinical severity* at the time of the scan (as measured by the PSPRS). The Cortical group showed strong bilateral positive correlations with multi-region cortical thickness and survival from scan but did not display symmetrical volumetric correlates with survival from scan. The Subcortical group showed strong positive bilateral frontal correlation between cortical thickness and survival from scan but did not display volumetric correlates with survival from scan. No significant symmetrical correlations between cortical area and survival from scan were observed in any group.

The multiple regression analysis of the predictive accuracy of survival from scan according to baseline characteristics and the 5 regions of interest is displayed in Supplementary Figure 4. In summary, the combination of left accumbens volume, left cerebellum volume, right insula thickness, left putamen volume and right putamen volume predicted 36% of the variance in survival time, from scan to death (*R^2^* = 0.364, *F*(8, 71) = 5.08, p<0.0001).

## Discussion

The principal result of this study is that atrophy in cortical and subcortical regions is related to how far ‘through’ the illness a patient with PSP is, from their symptom onset to death. We refer to this normalised survival as the ‘temporal stage’ of their PSP. These regional volume correlates differ from the baseline midbrain correlates of clinical severity (i.e., correlation with PSP Rating Scale). The structure-survival relationships also vary by clinical phenotype, with subcortical phenotypes demonstrating the strongest structure-survival relationships. Further, a multiple regression model constructed with just five regions of interest predicted the survival time from scan to death. Such an approach could in principle be used in clinical settings to support discussions about likely disease trajectories and prognostic estimates. Taken together, these findings suggest that despite the expected relationship between ‘temporal stage’ and clinical severity, their neuroanatomical correlates are distinct.

The concept of ‘temporal stage’ we use here warrants further discussion. We do not mean clinical severity staging, but the percentage in time of the disease course from onset to death. Unlike the absolute survival from scan date to date of death, this temporal staging normalizes the time of the imaging to the individual’s whole duration of symptomatic disease. This staging corrects for the marked variability in progression in PSP and allows severity of brain atrophy at a discrete timepoint to be compared with the elapsed fraction of the disease course at that point. We propose that temporal staging better accounts for heterogeneity than absolute time; indeed, ‘temporal stage’ had stronger correlations with regional volumes than ‘survival from scan’ in years. However, we recognise that ‘temporal stage’ is dependent on an estimate of the onset taken from the reported onset of symptoms, which may be biased with hindsight, and prone to error in patient and carer reports. It is likely that neuropathology onset precedes reported symptom onset by several years as it does in other forms of frontotemporal lobar degeneration.^46,49^ Similarly, the timing of death itself may be influenced by many factors, and not only reflect the extent of brain pathology. For example, environmental factors, home environment, daily care, medical care, and other comorbidities may also have an impact on survival from scan. In the UK, the National Health Service provides universal healthcare that is free at the point of care, including palliative care services which would be available in principle to all patients in our cohort, without dependence on personal wealth or insurance. In the UK, euthanasia and assisting access to euthanasia is illegal, and we are not aware of euthanasia in any of the cohort.

Note that we did not examine longitudinal changes in atrophy. Our outcome measure relates to the survival after imaging, not the volumetric change during the following year of life. Longitudinal imaging studies have shown significant progression of atrophy during one year, in regions cross-sectionally associated with PSP (e.g., the midbrain volume and medial frontal cortex), and at rates which differ from Parkinson’s disease.^17,27,50,51^ Decline in midbrain volumes correlate with changes in clinical measures.^27^ However, these studies address a very different question to that of the current study. Here we tested the relationship between brain structure and future survival, in other words, prediction. The anatomical correlates of survival are not the typical regions with most significant progressive atrophy or related to clinical severity.^52^ This disparity might arise from differential non-linear changes in volume between regions, or the specific range of measures included in the PSPRS.

The Richardson’s syndrome phenotype of PSP disrupts the circuits between orbitofrontal cortex, striatum and thalamus.^16,19,53–56^ Atrophy in these regions, and changes to white matter connections between them are well described in PSP, and likely underlie the functional breakdown in networks which support motor and cognitive functions.^52^ Our findings suggest that regional volume loss progresses in a largely symmetrical and linear pattern throughout the disease course in Richardson’s syndrome, with subcortical regions demonstrating a stronger linear relationship than cortical regions. In typical Richardson’s syndrome, subcortical regions develop neuroglial pathology first, and may therefore have more advanced degeneration compared to cortical areas at mid and later stages of disease.^57^ This may increase their predictive signal-to-noise. The globular rather than sheet-like morphology of many subcortical regions (e.g., thalamus, pallidum), may also increase the volumetric signal-to-noise ratio. Changes in the intrinsic grey matter signal, and juxta-cortical white matter, from PSP pathology might in principle change the signal intensities that affect the volume estimations, thus contributing to the findings in our study.

Subcortical phenotypes demonstrated stronger linear relationships between temporal stage and regional volumes than Richardson’s syndrome while consistent, symmetrical findings were not demonstrated in Cortical phenotypes. It may be that the heterogeneity of the Cortical phenotypes, and low numbers in comparison to the Richardson’s syndrome group, contributed to reduced power and thus Type II error. If the null result is true, rather than Type II error, it would suggest (but not in itself prove) that the PSPRS is a function of cortical pathology. This is plausible, as many of the actions and tasks referred to in the PSPRS are based on cortical systems (e.g., cognition, social engagement, behaviour, praxis, volitional motor and oculomotor control), and change in PSPRS over a year of PSP disease progression correlate with cortical synaptic loss.^58^ Encouragingly, the strongest relationships between temporal stage and regional volumes were shown in the group with the highest degree of diagnostic certainty; those with neuropathological confirmation. This study was not designed to identify the mediating factors in survival, but the structural data raise hypotheses about the determinants and predictors of survival which require further testing in larger and prospective cohorts.

This study has several limitations. The date of symptom onset is challenging to estimate, especially for Cortical phenotypes: the onset of motor symptoms or a first fall may be clearly recalled but subtler changes in behaviour or personality may not be. Indeed, common cognitive and behavioural changes such as apathy, impulsivity, language and executive dysfunction are frequently missed or underestimated in early stages of the disease^59–64^ while the MAX rules^45^ are intrinsically based towards Richardson’s syndrome’s motor features as the illness progresses from variant phenotypes. This lack of clarity will affect the estimation of survival from onset to death and hence the calculation of temporal ‘stage’. Retrospective allocation of phenotypes is also subject to error, especially when reliant on contemporaneous clinical notes. A clinician practicing in 2007, for example, may have been more likely to have documented features integral to the diagnostic criteria of the time, rather than highlighting characteristics suggestive of a variant phenotype by today’s standards. Whilst the cross-sectional nature of the study allows correlation with survival, it lacks the longitudinal imaging necessary for formal mediation analysis. In part this reflects the long timespan of the current study (2007-2019), during which time there have been marked changes in diagnosis, phenotyping and MRI sequences and scanners. Our analysis utilised T1-weighted volumetric imaging building on the established literature of grey and white matter changes in PSP, but our inferences about networks are based on overlapping spatial distributions rather than functional imaging which would provide further valuable information.

In summary, atrophy of a symmetric network of subcortical regions in the striatum, cerebellum and frontotemporal cortex is related to survival risk in PSP, manifest as a correlation with the temporal stage from symptom onset to death. The relationship between atrophy and survival may be useful to identify and treat the mechanisms of poor survival, and to stratify heterogeneous patient cohorts in clinical trials with optimised outcome measures for disease-modifying approaches.

## Supplementary Material

Supplementary Material

## Figures and Tables

**Table 1A:** Clinical and demographic characteristics of the study population split by disease phenotype

	PSP-RS	PSP-C	PSP-SC	PSP-C vs PSP-RS	PSP-C vs PSP-SC	PSP-RS vs PSP-SC
	N=80	N=16	N=16	p-value	p-value	p-value
Sex, Male/Female (%)	0.60 (0.49)	0.56 (0.51)	0.62 (0.50)	0.959	0.933	0.982
Age at symptom onset	67.3 (7.22)	67.0 (9.11)	64.6 (7.73)	0.986	0.642	0.387
Age at diagnosis	70.6 (6.76)	70.4 (8.28)	70.5 (6.29)	0.995	1.000	0.998
Age at scan	71.2 (6.75)	71.6 (7.96)	70.6 (6.43)	0.968	0.902	0.947
Age at death	73.7 (6.54)	73.9 (7.25)	73.6 (6.67)	0.991	0.990	0.999
Duration from symptom onset to diagnosis	3.29 (2.34)	3.44 (2.34)	5.92 (4.24)	0.701	0.171	0.019
Duration from symptom onset to scan	3.86 (2.39)	4.65 (2.63)	6.01 (4.20)	0.150	0.670	0.140
Total disease duration (symptom onset to death)	6.38 (2.63)	6.94 (3.73)	9.04 (5.09)	0.980	0.420	0.210
Duration from diagnosis to death	3.09 (1.56)	3.50 (2.65)	3.12 (1.77)	0.960	0.870	0.760
Survival from scan	2.52 (1.53)	2.29 (1.73)	3.03 (1.94)	0.450	0.420	0.470
Temporal Stage	0.60 (0.20)	0.68 (0.19)	0.63 (0.14)	0.289	0.758	0.823
PSP Rating Scale	37.1 (13.1)	32.9 (17.3)	39.0 (10.8)	0.704	0.603	0.895
Scan to PSP Rating Scale (days)	26.7 (28.8)	29.3 (31.5)	29.6 (25.4)	0.738	0.854	0.626
Phenotype						
PSP-CBS	0	7 (44%)	0			
PSP-F	0	6 (37%)	0			
PSP-P	0	0	7 (44%)			
PSP-PGF	0	0	3 (19%)			
PSP-PI	0	0	6 (37%)			
PSP-RS	80 (100%)	0	0			
PSP-SL	0	3 (19%)	0			

Abbreviations: PSP = progressive supranuclear palsy, PSP-C = PSP-Cortical, PSP-CBS = PSP corticobasal syndrome, PSP-F = PSP frontal, PSP-P = PSP parkinsonism, PSP-PGF = PSP progressive gait freezing, PSP-PI = PSP postural instability, PSP-RS = PSP-Richardson’s syndrome, PSP-SC = PSP-Subcortical, PSP-SL = speech and language.

**Table 1B:** Clinical and demographic characteristics of the study population with pathological confirmation of diagnosis split by disease phenotype

	PSP-RS	PSP-C	PSP-SC	PSP-C vs PSP-RS	PSP-C vs PSP-SC	PSP-RS vs PSP-SC
	N=30	N=12	N=7	p-value	p-value	p-value
Sex, Male/Female (%)	0.53 (0.51)	0.50 (0.52)	0.43 (0.53)	1.000	1.000	1.000
Age at symptom onset	65.8 (6.85)	67.8 (9.52)	62.7 (8.29)	0.735	0.362	0.613
Age at diagnosis	69.4 (6.97)	70.8 (9.09)	68.7 (6.74)	0.843	0.824	0.974
Age at scan	70.0 (6.95)	72.2 (8.64)	69.1 (6.96)	0.657	0.657	0.958
Age at death	72.7 (6.44)	74.5 (7.77)	71.4 (6.34)	0.715	0.602	0.892
Duration from symptom onset to diagnosis	3.62 (2.53)	3.06 (1.77)	6.02 (4.96)	0.630	0.260	0.220
Duration from symptom onset to scan	4.22 (2.79)	4.45 (2.34)	6.44 (5.31)	0.520	0.540	0.530
Total disease duration (symptom onset to death)	6.91 (2.57)	6.73 (3.74)	8.71 (5.26)	0.210	0.650	0.690
Duration from diagnosis to death	3.29 (1.66)	3.68 (3.01)	2.68 (1.48)	0.750	0.650	0.340
Survival from scan	2.69 (1.59)	2.29 (1.84)	2.26 (1.34)	0.280	1.000	0.490
Temporal Stage	0.60 (0.20)	0.67 (0.18)	0.67 (0.17)	0.566	0.998	0.642
PSP Rating Scale	39.0 (15.2)	38.4 (17.5)	43.0 (14.9)	0.997	0.914	0.910
Scan to PSP Rating Scale (days)	21.3 (22.8)	35.8 (34.9)	35.0 (35.0)	0.336	0.881	0.517
Phenotype						
PSP-CBS	0	6 (50%)	0			
PSP-F	0	3 (25%)	0			
PSP-P	0	0	2 (29%)			
PSP-PGF	0	0	1 (14%)			
PSP-PI	0	0	4 (57%)			
PSP-RS	30 (100%)	0	0			
PSP-SL	0	3 (25%)	0			

Abbreviations: PSP = progressive supranuclear palsy, PSP-C = PSP-Cortical, PSP-CBS = PSP corticobasal syndrome, PSP-F = PSP frontal, PSP-P = PSP parkinsonism, PSP-PGF = PSP progressive gait freezing, PSP-PI = PSP postural instability, PSP-RS = PSP-Richardson’s syndrome, PSP-SC = PSP-Subcortical, PSP-SL = speech and language.

**Table 2:** Within group summary table of top 5 strongest Pearson’s r correlations between region-of-interest parameters and Progressive Supranuclear Palsy Rating Scale (PSP-RS), temporal stage and survival from scan

	All (*n*=80)			Path (*n*=28)			Richardson’s Syndrome (*n*=62)			Cortical (*n*=7)			Subcortical (*n*=11)		
Marker	Region	r	p-value	Region	r	p-value	Region	r	p-value	Region	r	p-value	Region	r	p-value
**PSP-RS**															
	Left frontal area	-0.351	<0.001	Left frontal area	-0.653	<0.001	Left cerebellum volume	-0.392	<0.001	Left frontal area	-0.756	0.026	Left temporal thickness	-0.764	<0.001
	Left cerebellum volume	-0.340	<0.001	Left ventral diencephalon volume	-0.648	<0.001	Right cerebellum volume	-0.375	<0.001	Left frontal volume	-0.752	0.036	Left insula volume	-0.666	0.009
	Right cerebellum volume	-0.321	<0.001	Left thalamus volume	-0.643	<0.001	Midbrain volume	-0.325	0.002	Right cingulate area	-0.734	0.026	Right cingulate volume	-0.640	0.009
	Right cingulate area	-0.316	<0.001	Left temporal area	-0.625	<0.001	Right cingulate area	-0.319	0.004	Left temporal area	-0.679	0.043	Right frontal volume	-0.629	0.009
	Right frontal area	-0.314	<0.001	Left putamen volume	-0.618	<0.001	Superior cerebellar peduncle volume	-0.294	0.006	Right frontal volume	-0.629	0.088	Left frontal volume	-0.613	0.009
**Temporal Stage**															
	Left accumbens volume	-0.328	<0.001	Left accumbens volume	-0.545	<0.001	Left accumbens volume	-0.284	0.002	Left occipital thickness	-0.858	<0.001	Right putamen volume	-0.754	<0.001
	Left amygdala volume	-0.217	0.009	Left temporal thickness	-0.402	0.002	Left amygdala volume	-0.264	0.002	Left parietal thickness	-0.806	<0.001	Right parietal thickness	-0.690	<0.001
	Left cerebellum volume	-0.214	0.008	Left temporal volume	-0.401	0.002	Left cerebellum volume	-0.217	0.012	Left frontal thickness	-0.765	<0.001	Right ventral diencephalon volume	-0.682	<0.001
	Right putamen volume	-0.200	0.012	Right temporal thickness	-0.386	0.002	Right putamen volume	-0.202	0.020	Right frontal thickness	-0.705	0.001	Left occipital volume	-0.648	<0.001
	Left putamen volume	-0.194	0.012	Left putamen volume	-0.381	0.003	Left putamen volume	-0.191	0.029	Right occipital thickness	-0.700	0.001	Right accumbens volume	-0.625	0.002
**Survival from Scan**															
	Left accumbens volume	0.353	<0.001	Left accumbensvolume	0.538	<0.001	Left accumbens volume	0.397	<0.001	Right frontal thickness	0.779	<0.001	Right frontal thickness	0.677	<0.001
	Left cerebellum volume	0.253	<0.001	Right insula thickness	0.372	0.007	Left putamen volume	0.311	<0.001	Left temporal thickness	0.755	<0.001	Right insula thickness	0.546	0.005
	Right insula thickness	0.251	0.001	Left temporal thickness	0.350	0.007	Left cerebellum volume	0.288	<0.001	Left parietal thickness	0.709	0.002	Left frontal thickness	0.544	0.005
	Left putamen volume	0.247	0.001	Left putamen volume	0.305	0.024	Left amygdala volume	0.276	<0.001	Left frontal thickness	0.690	0.002	Left insula thickness	0.398	0.072
	Right putamen volume	0.210	0.005	Right temporal thickness	0.290	0.031	Left ventral diencephalon volume	0.266	<0.001	Right insula thickness	0.655	0.004	Left parietal thickness	0.366	0.081
