# Supplementary material for: Structural correlates of survival in Progressive Supranuclear Palsy
Source: Parkinsonism Relat Disord. Author manuscript; Available in PMC 2023 Oct 19. (PMC7615224; doi:10.1016/j.parkreldis.2023.105866)
Supplement: Supplementary Material [file EMS188476-supplement-Supplementary_Material.docx]

**Supplementary Figure 1: Structural correlates of severity.** Pearson’s r partial correlation coefficient analysis between Progressive Supranuclear Palsy Rating Scale and region-of-interest volumes. Significant of relationship is illustrated by log-transformed false discovery rate corrected p-values from partial correlation analysis. RS = Richardson’s syndrome.

*
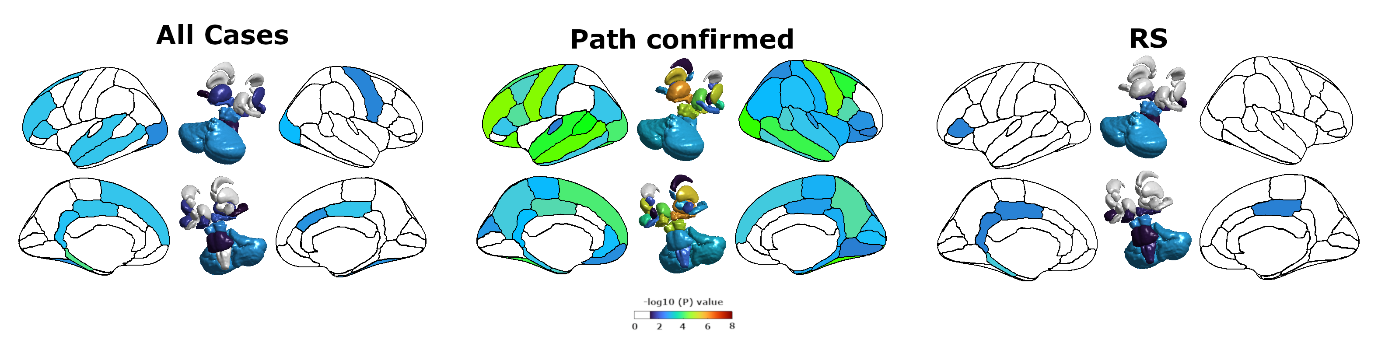
*

**Supplementary Figure 2: Structural correlates of survival in terms of temporal stage (percent from onset to death) and survival from scan.** Pearson’s r partial correlation coefficient analysis between stage, survival from scan (SFS) and region-of-interest volumes, split by all cases and those with pathological confirmation. Significance of relationship is illustrated by log-transformed false discovery rate corrected p-values from partial correlation analysis**.**


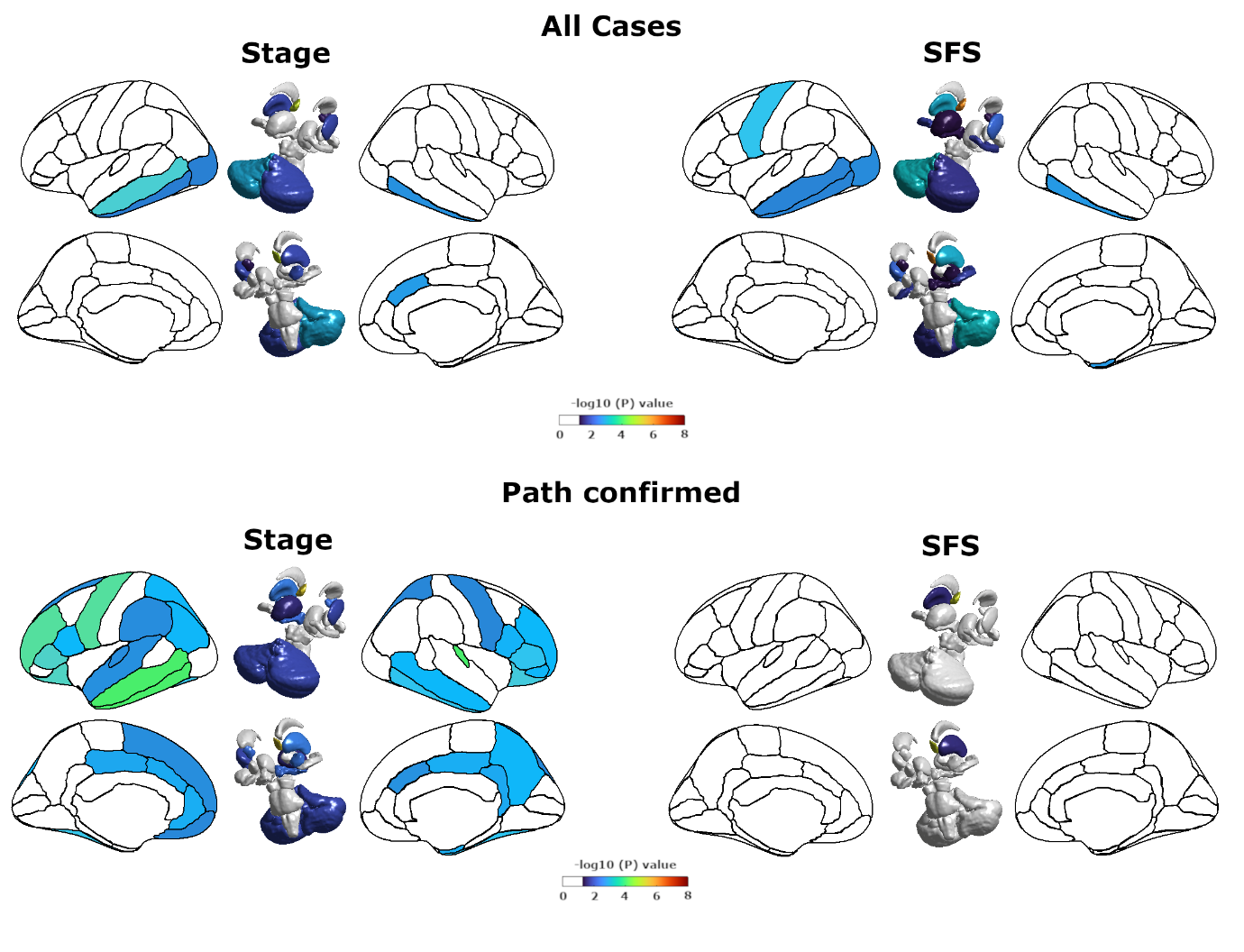


**Supplementary Figure 3: Structural correlates of temporal stage and survival from scan compared across diagnostic groups.** Fisher’s one-tail Z-test comparison between Pearson’s r partial correlation coefficients of stage, survival from scan (SFS) and subcortical region-of-interest volumes, split by phenotype during life. Significance of relationship is illustrated by log-transformed false discovery rate corrected p-values from Fisher’s one tail Z-test. RS = Richardson’s syndrome.


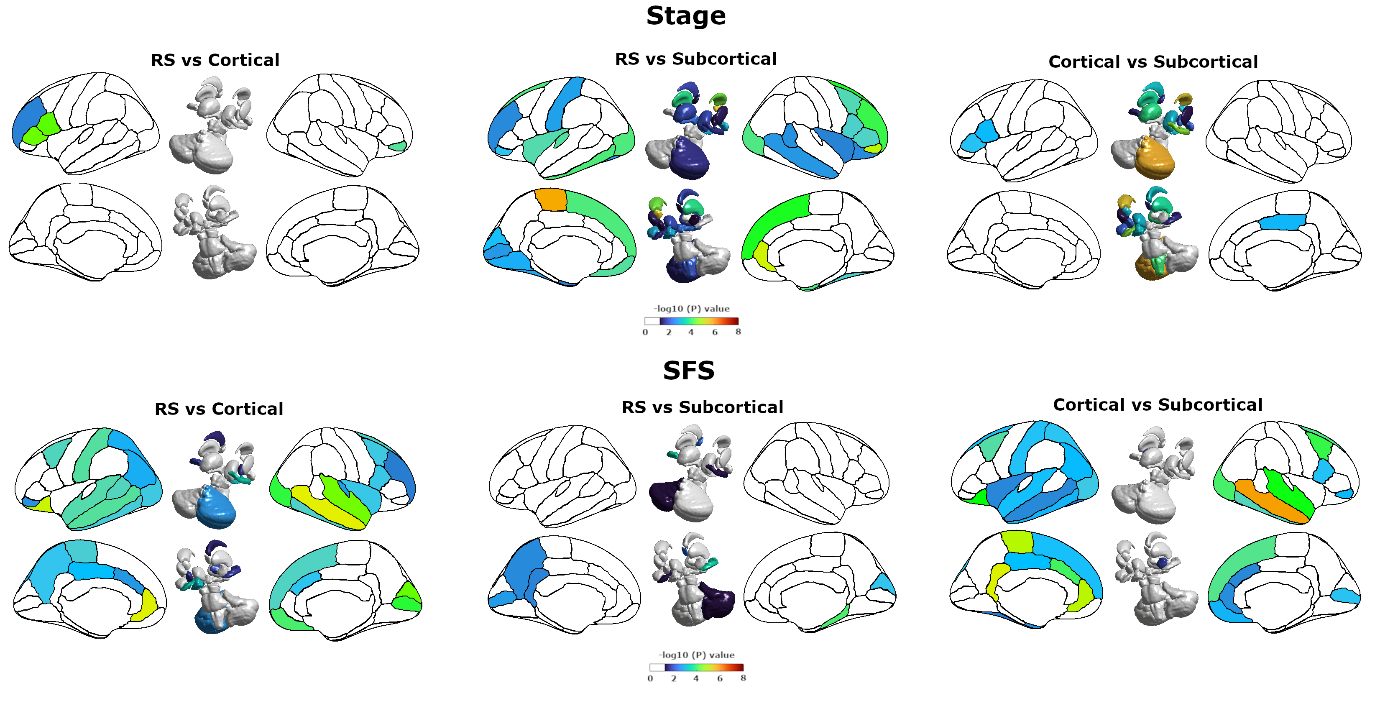


**Supplementary Figure 4: Accuracy of survival from scan prediction.** Scatter plot of actual versus predicted values calculated according to construction of a multiple regression model constituting the top 5 region-of-interest parameters and participant characteristics.


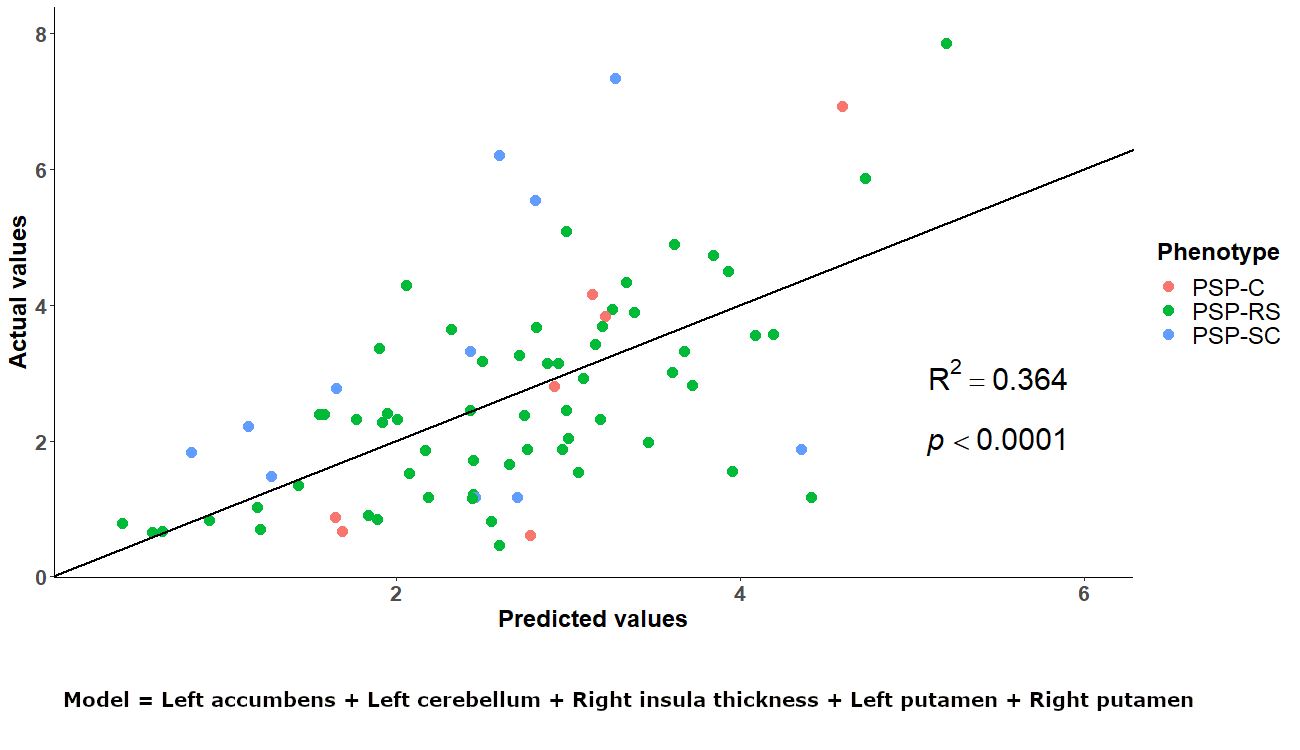


**Supplementary Table 15: Within group Pearson’s r partial correlation coefficients between Progressive Supranuclear Palsy Rating Scale and region-of-interest volumes**

| **Region** | **All (n = 80)** | | **Path (n = 28)** | | **RS (n = 62)** | | **Cortical (n = 7)** | | **Subcortical (n = 11)** | |
| --- | --- | --- | --- | --- | --- | --- | --- | --- | --- | --- |
|  | **R** | **P-value** | **R** | **P-value** | **R** | **P-value** | **R** | **P-value** | **R** | **P-value** |
| **Left Thalamus** | -0.226 | 0.017 | -0.643 | <0.001 | -0.176 | 0.122 | -0.527 | 0.271 | -0.555 | 0.156 |
| **Left Caudate** | 0.070 | 0.436 | -0.288 | 0.046 | 0.066 | 0.551 | -0.223 | 0.661 | 0.097 | 0.756 |
| **Left Putamen** | -0.180 | 0.056 | -0.618 | <0.001 | -0.136 | 0.240 | -0.509 | 0.287 | -0.315 | 0.318 |
| **Left Pallidum** | -0.164 | 0.086 | -0.456 | 0.001 | -0.121 | 0.304 | -0.228 | 0.661 | -0.433 | 0.189 |
| **Left Hippocampus** | -0.186 | 0.049 | -0.450 | 0.001 | -0.147 | 0.209 | -0.390 | 0.475 | -0.420 | 0.201 |
| **Left Amygdala** | -0.129 | 0.166 | -0.374 | 0.009 | -0.157 | 0.187 | 0.373^a^ | 0.498 | -0.456 | 0.168 |
| **Left Accumbens** | -0.116 | 0.206 | -0.440 | 0.002 | -0.077 | 0.484 | -0.413 | 0.438 | -0.222 | 0.506 |
| **Left Ventral DC** | -0.249 | 0.007 | -0.648 | <0.001 | -0.245 | 0.029 | -0.317 | 0.554 | -0.364 | 0.306 |
| **Left Cerebellum** | -0.340 | <0.001 | -0.529 | <0.001 | -0.392 | <0.001 | -0.256 | 0.399 | -0.082 | 0.786 |
| **Right Thalamus** | -0.140 | 0.136 | -0.553 | <0.001 | -0.130^a^ | 0.262 | -0.143 | 0.785 | -0.548 | 0.156 |
| **Right Caudate** | 0.089 | 0.350 | -0.056 | 0.753 | 0.083 | 0.475 | -0.170 | 0.732 | 0.112 | 0.725 |
| **Right Putamen** | -0.216 | 0.018 | -0.601 | <0.001 | -0.180 | 0.119 | -0.537 | 0.271 | -0.477 | 0.156 |
| **Right Pallidum** | -0.130 | 0.166 | -0.295 | 0.042 | -0.111^a^ | 0.335 | 0.125^a^ | 0.785 | -0.510 | 0.156 |
| **Right Hippocampus** | -0.117 | 0.206 | -0.413 | 0.003 | -0.080 | 0.481 | -0.447 | 0.397 | -0.322 | 0.318 |
| **Right Amygdala** | -0.220 | 0.018 | -0.519 | <0.001 | -0.219 | 0.044 | -0.228 | 0.661 | -0.452 | 0.168 |
| **Right Accumbens** | -0.089 | 0.350 | -0.368 | 0.009 | -0.045^a^ | 0.697 | -0.106 | 0.785 | -0.514 | 0.156 |
| **Right Ventral DC** | -0.218 | 0.018 | -0.603 | <0.001 | -0.230 | 0.036 | -0.325 | 0.554 | -0.345 | 0.318 |
| **Right Cerebellum** | -0.321 | <0.001 | -0.509 | <0.001 | -0.375 | <0.001 | -0.336 | 0.399 | -0.063 | 0.786 |
|  |  |  |  |  |  |  |  |  |  |  |
| **Right Frontal** | -0.193 | 0.025 | -0.517 | <0.001 | -0.126 | 0.247 | -0.629^c^ | 0.088 | -0.629^d^ | 0.009 |
| **Right Parietal** | -0.130 | 0.135 | -0.424 | 0.003 | -0.119 | 0.252 | -0.339 | 0.461 | -0.297 | 0.192 |
| **Right Temporal** | -0.183 | 0.032 | -0.536 | <0.001 | -0.128 | 0.247 | -0.418 | 0.372 | -0.548^c^ | 0.020 |
| **Right Occipital** | -0.222 | 0.015 | -0.505 | <0.001 | -0.173 | 0.133 | -0.331 | 0.461 | -0.503 | 0.027 |
| **Right Cingulate** | -0.236 | 0.011 | -0.320 | 0.019 | -0.216 | 0.133 | -0.223 | 0.619 | -0.640^c^ | 0.009 |
| **Right Insula** | -0.061 | 0.444 | -0.353 | 0.010 | -0.047 | 0.730 | 0.085 | 0.783 | -0.441^c^ | 0.054 |
| **Left Frontal** | -0.265 | 0.006 | -0.598 | <0.001 | -0.182 | 0.133 | -0.752^d^ | 0.036 | -0.613^c^ | 0.009 |
| **Left Parietal** | -0.074 | 0.387 | -0.407 | 0.003 | -0.033 | 0.779 | -0.086 | 0.783 | -0.503^c^ | 0.027 |
| **Left Temporal** | -0.259 | 0.006 | -0.578 | <0.001 | -0.189 | 0.133 | -0.626^c^ | 0.088 | -0.597^c^ | 0.010 |
| **Left Occipital** | -0.201 | 0.023 | -0.413 | 0.003 | -0.146 | 0.214 | -0.465 | 0.329 | -0.397 | 0.081 |
| **Left Cingulate** | -0.200 | 0.023 | -0.402 | 0.003 | -0.186 | 0.133 | -0.163 | 0.713 | -0.537^c^ | 0.021 |
| **Left Insula** | -0.084 | 0.354 | -0.215 | 0.115 | -0.012 | 0.892 | -0.302 | 0.474 | -0.666^d^ | 0.009 |
|  |  |  |  |  |  |  |  |  |  |  |
| **Medulla** | -0.144 | 0.128 | -0.400 | 0.005 | -0.236 | 0.033 | 0.670^b^ | 0.116 | -0.305 | 0.324 |
| **Pons** | -0.194 | 0.039 | -0.451 | 0.001 | -0.221 | 0.044 | 0.197^a^ | 0.679 | -0.481 | 0.156 |
| **SCP** | -0.217 | 0.018 | -0.126 | 0.429 | -0.294 | 0.006 | 0.114 | 0.785 | -0.255 | 0.437 |
| **Midbrain** | -0.286 | 0.002 | -0.587 | <0.001 | -0.325 | 0.002 | 0.072 | 0.837 | -0.490 | 0.156 |
| Abbreviations: PSP = Progressive supranuclear palsy, R = Pearson’s R partial correlation coefficient, Path = neuropathologically confirmed cases, RS = Richardson’s syndrome, DC = diencephalon, SCP = superior cerebellar peduncle.   1. Fisher’s one-tail Z-test p<0.05 vs Subcortical group 2. Fisher’s one-tail Z-test p<0.01 vs Subcortical group 3. Fisher’s one-tail Z-test p<0.05 vs Richardson’s syndrome group 4. Fisher’s one-tail Z-test p<0.01 vs Richardson’s syndrome group | | | | | | | | | | |

**Supplementary Table 26: Within group Pearson’s r partial correlation coefficients between temporal ‘stage’ and region-of-interest volumes**

| **Region** | **All (n = 80)** | | **Path (n = 28)** | | **RS (n = 62)** | | **Cortical (n = 7)** | | **Subcortical (n = 11)** | |
| --- | --- | --- | --- | --- | --- | --- | --- | --- | --- | --- |
|  | **R** | **P-value** | **R** | **P-value** | **R** | **P-value** | **R** | **P-value** | **R** | **P-value** |
| **Left Thalamus** | -0.074 | 0.459 | -0.277 | 0.025 | -0.117^a^ | 0.210 | 0.223^c^ | 0.527 | -0.253 | 0.248 |
| **Left Caudate** | 0.034 | 0.788 | -0.057 | 0.681 | 0.089^a^ | 0.366 | 0.621^b^ | 0.029 | -0.343 | 0.109 |
| **Left Putamen** | -0.194 | 0.012 | -0.381 | 0.003 | -0.191^c^ | 0.029 | 0.319^c^ | 0.315 | -0.562 | 0.006 |
| **Left Pallidum** | 0.108 | 0.216 | -0.147 | 0.244 | 0.178^a^ | 0.041 | 0.479 | 0.124 | -0.486 | 0.023 |
| **Left Hippocampus** | -0.071 | 0.475 | -0.174 | 0.177 | -0.088^a^ | 0.366 | 0.254^b^ | 0.463 | -0.440 | 0.037 |
| **Left Amygdala** | -0.217 | 0.009 | -0.370 | 0.004 | -0.264 | 0.002 | 0.311^a^ | 0.315 | -0.267 | 0.230 |
| **Left Accumbens** | -0.328 | <0.001 | -0.545 | <0.001 | -0.284^a^ | 0.002 | -0.543 | 0.063 | -0.551 | 0.007 |
| **Left Ventral DC** | -0.095 | 0.298 | -0.354 | 0.005 | -0.116^a^ | 0.210 | 0.170^a^ | 0.665 | -0.440 | 0.037 |
| **Left Cerebellum** | -0.214 | 0.002 | -0.297 | 0.006 | -0.217 | 0.006 | -0.312 | 0.290 | -0.331 | 0.130 |
| **Right Thalamus** | <0.001 | 0.996 | -0.146 | 0.244 | -0.032^a^ | 0.786 | 0.742^c^ | 0.007 | -0.405 | 0.051 |
| **Right Caudate** | -0.044 | 0.700 | 0.027 | 0.833 | -0.008^a^ | 0.915 | 0.451^b^ | 0.134 | -0.429 | 0.039 |
| **Right Putamen** | -0.200 | 0.012 | -0.315 | 0.012 | -0.202^c^ | 0.020 | 0.346^c^ | 0.269 | -0.754 | <0.001 |
| **Right Pallidum** | 0.206 | 0.012 | 0.036 | 0.789 | 0.235^a^ | 0.006 | 0.382^a^ | 0.228 | -0.120 | 0.608 |
| **Right Hippocampus** | 0.002 | 0.996 | -0.157 | 0.228 | -0.025^a^ | 0.795 | 0.361^b^ | 0.263 | -0.362 | 0.093 |
| **Right Amygdala** | -0.062 | 0.564 | -0.196 | 0.137 | -0.075^a^ | 0.455 | 0.617^c^ | 0.029 | -0.444 | 0.037 |
| **Right Accumbens** | -0.165 | 0.037 | -0.354 | 0.005 | -0.126^b^ | 0.186 | 0.003^b^ | 0.990 | -0.625 | 0.002 |
| **Right Ventral DC** | -0.007 | 0.993 | -0.226 | 0.080 | 0.052^c^ | 0.606 | 0.117^b^ | 0.763 | -0.682 | <0.001 |
| **Right Cerebellum** | -0.176 | 0.006 | -0.346 | 0.003 | -0.186 | 0.011 | -0.248 | 0.290 | -0.183 | 0.317 |
|  |  |  |  |  |  |  |  |  |  |  |
| **Right Frontal** | -0.005 | 0.976 | -0.303 | 0.011 | 0.026^b^ | 0.795 | 0.299 | 0.402 | -0.531 | 0.007 |
| **Right Parietal** | -0.097 | 0.236 | -0.258 | 0.028 | -0.075 | 0.413 | -0.147 | 0.716 | -0.153 | 0.440 |
| **Right Temporal** | -0.116 | 0.214 | -0.345 | 0.004 | -0.102^c^ | 0.310 | 0.417 | 0.340 | -0.468 | 0.017 |
| **Right Occipital** | -0.110 | 0.214 | -0.204 | 0.071 | -0.126^b^ | 0.223 | 0.592 | 0.072 | -0.556 | 0.006 |
| **Right Cingulate** | -0.183 | 0.043 | -0.346 | 0.004 | -0.124 | 0.223 | -0.303 | 0.402 | -0.338 | 0.078 |
| **Right Insula** | -0.002 | 0.976 | -0.222 | 0.058 | 0.032^c^ | 0.795 | 0.089 | 0.815 | -0.296 | 0.120 |
| **Left Frontal** | -0.047 | 0.567 | -0.374 | 0.003 | 0.003^c^ | 0.963 | -0.031 | 0.897 | -0.420 | 0.028 |
| **Left Parietal** | -0.096 | 0.236 | -0.324 | 0.007 | -0.093 | 0.310 | 0.309 | 0.402 | -0.394 | 0.039 |
| **Left Temporal** | -0.152 | 0.075 | -0.401 | 0.002 | -0.135^c^ | 0.223 | 0.203 | 0.587 | -0.475 | 0.017 |
| **Left Occipital** | -0.173 | 0.043 | -0.209 | 0.070 | -0.131^b^ | 0.223 | 0.077 | 0.815 | -0.648 | 0.001 |
| **Left Cingulate** | -0.080 | 0.326 | -0.298 | 0.011 | -0.094 | 0.310 | 0.395 | 0.340 | -0.138 | 0.453 |
| **Left Insula** | 0.048 | 0.567 | -0.159 | 0.151 | 0.136^b^ | 0.223 | -0.238 | 0.537 | -0.424 | 0.028 |
|  |  |  |  |  |  |  |  |  |  |  |
| **Medulla** | -0.085 | 0.376 | -0.236 | 0.067 | -0.114 | 0.214 | 0.631^c^ | 0.029 | -0.323 | 0.136 |
| **Pons** | -0.014 | 0.955 | -0.176 | 0.176 | -0.010^b^ | 0.913 | 0.551^c^ | 0.063 | -0.454 | 0.037 |
| **SCP** | 0.002 | 0.996 | 0.096 | 0.460 | -0.056 | 0.591 | 0.131 | 0.762 | -0.040 | 0.849 |
| **Midbrain** | -0.026 | 0.870 | -0.208 | 0.113 | -0.025^b^ | 0.795 | 0.616^c^ | 0.029 | -0.572 | 0.006 |
| Abbreviations: PSP = Progressive supranuclear palsy, R = Pearson’s R partial correlation coefficient, Path = neuropathologically confirmed cases, RS = Richardson’s syndrome, DC = diencephalon, SCP = superior cerebellar peduncle.   1. Fisher’s one-tail Z-test p<0.05 vs Subcortical group 2. Fisher’s one-tail Z-test p<0.01 vs Subcortical group 3. Fisher’s one-tail Z-test p<0.001 vs Subcortical group | | | | | | | | | | |

**Supplementary Table 37: Within group Pearson’s r partial correlation coefficients between survival from scan and region-of-interest volumes**

| **Region** | **All (n = 80)** | | **Path (n = 28)** | | **RS (n = 62)** | | **Cortical (n = 7)** | | **Subcortical (n = 11)** | |
| --- | --- | --- | --- | --- | --- | --- | --- | --- | --- | --- |
|  | **R** | **P-value** | **R** | **P-value** | **R** | **P-value** | **R** | **P-value** | **R** | **P-value** |
| **Left Thalamus** | 0.149 | 0.042 | 0.114 | 0.445 | 0.211 | 0.009 | -0.384 | 0.240 | 0.040 | 0.899 |
| **Left Caudate** | 0.014 | 0.872 | -0.057 | 0.720 | -0.032^d^ | 0.717 | -0.342 | 0.281 | 0.200 | 0.740 |
| **Left Putamen** | 0.247 | 0.001 | 0.305 | 0.024 | 0.311 | <0.001 | -0.152 | 0.640 | 0.229 | 0.740 |
| **Left Pallidum** | -0.160 | 0.034 | 0.009 | 0.934 | -0.118 | 0.165 | -0.403 | 0.216 | -0.184 | 0.740 |
| **Left Hippocampus** | 0.187 | 0.014 | 0.157 | 0.322 | 0.265^d^ | 0.001 | -0.273 | 0.421 | -0.058^g^ | 0.899 |
| **Left Amygdala** | 0.153 | 0.040 | 0.263 | 0.070 | 0.276^d^ | 0.001 | -0.242 | 0.447 | -0.373^i^ | 0.590 |
| **Left Accumbens** | 0.353 | <0.001 | 0.538 | <0.001 | 0.397 | <0.001 | 0.640^a^ | 0.030 | 0.104 | 0.855 |
| **Left Ventral DC** | 0.154 | 0.040 | 0.251 | 0.084 | 0.266 | 0.001 | -0.065 | 0.785 | -0.248^h^ | 0.740 |
| **Left Cerebellum** | 0.253 | <0.001 | 0.250 | 0.046 | 0.288 | <0.001 | 0.321 | 0.335 | 0.083 | 0.974 |
| **Right Thalamus** | 0.114 | 0.125 | 0.075 | 0.637 | 0.145^e^ | 0.092 | -0.519 | 0.103 | 0.059 | 0.899 |
| **Right Caudate** | 0.041 | 0.663 | -0.090 | 0.570 | 0.054 | 0.551 | -0.262 | 0.422 | 0.106 | 0.855 |
| **Right Putamen** | 0.210 | 0.005 | 0.141 | 0.366 | 0.242 | 0.002 | -0.128 | 0.678 | 0.334 | 0.590 |
| **Right Pallidum** | -0.181 | 0.016 | -0.107 | 0.472 | -0.192 | 0.019 | -0.358 | 0.272 | -0.054 | 0.899 |
| **Right Hippocampus** | 0.182 | 0.016 | 0.069 | 0.659 | 0.229^d^ | 0.004 | -0.278 | 0.421 | 0.113 | 0.855 |
| **Right Amygdala** | 0.019 | 0.839 | -0.025 | 0.893 | 0.123^f^ | 0.158 | -0.612 | 0.037 | -0.220^g^ | 0.740 |
| **Right Accumbens** | 0.149 | 0.042 | 0.168 | 0.322 | 0.182 | 0.028 | 0.187 | 0.547 | 0.044 | 0.899 |
| **Right Ventral DC** | 0.098 | 0.201 | 0.159 | 0.322 | 0.114 | 0.173 | 0.073 | 0.779 | -0.096 | 0.855 |
| **Right Cerebellum** | 0.203 | 0.002 | 0.218 | 0.048 | 0.245 | <0.001 | 0.171 | 0.470 | 0.006 | 0.974 |
|  |  |  |  |  |  |  |  |  |  |  |
| **Right Frontal** | 0.042 | 0.618 | -0.007 | 0.999 | 0.025^f^ | 0.734 | -0.720^c^ | 0.002 | 0.243 | 0.532 |
| **Right Parietal** | 0.151 | 0.085 | 0.067 | 0.938 | 0.133 | 0.144 | 0.032 | 0.961 | 0.280 | 0.532 |
| **Right Temporal** | 0.149 | 0.085 | 0.102 | 0.938 | 0.173^f^ | 0.070 | -0.702^c^ | 0.002 | 0.299 | 0.532 |
| **Right Occipital** | 0.058 | 0.557 | -0.042 | 0.938 | 0.153^f^ | 0.090 | -0.592^b^ | 0.018 | 0.169 | 0.532 |
| **Right Cingulate** | 0.074 | 0.504 | 0.126 | 0.938 | 0.123 | 0.163 | -0.104 | 0.793 | 0.093 | 0.613 |
| **Right Insula** | -0.009 | 0.890 | -0.017 | 0.999 | 0.061^e^ | 0.491 | -0.511 | 0.050 | -0.093 | 0.613 |
| **Left Frontal** | 0.064 | 0.550 | 0.060 | 0.938 | 0.054^d^ | 0.510 | -0.370^a^ | 0.146 | 0.205 | 0.532 |
| **Left Parietal** | 0.095 | 0.343 | 0.049 | 0.938 | 0.170^e^ | 0.070 | -0.389^a^ | 0.146 | 0.115 | 0.613 |
| **Left Temporal** | 0.144 | 0.085 | 0.152 | 0.938 | 0.195^e^ | 0.070 | -0.499^b^ | 0.050 | 0.177 | 0.532 |
| **Left Occipital** | 0.142 | 0.085 | 0.059 | 0.938 | 0.167 | 0.070 | -0.012 | 0.961 | 0.180 | 0.532 |
| **Left Cingulate** | 0.045 | 0.618 | <0.001 | 0.999 | 0.112^f^ | 0.193 | -0.711^c^ | 0.002 | 0.268 | 0.532 |
| **Left Insula** | -0.037 | 0.618 | -0.082 | 0.938 | -0.061 | 0.491 | -0.369 | 0.146 | 0.136 | 0.609 |
|  |  |  |  |  |  |  |  |  |  |  |
| **Medulla** | 0.135 | 0.064 | 0.205 | 0.171 | 0.255 | 0.001 | -0.145 | 0.643 | -0.284^h^ | 0.740 |
| **Pons** | 0.086 | 0.271 | 0.157 | 0.322 | 0.121 | 0.158 | -0.218 | 0.502 | -0.105 | 0.855 |
| **SCP** | 0.027 | 0.800 | -0.124 | 0.402 | 0.041 | 0.650 | -0.327 | 0.302 | 0.022 | 0.930 |
| **Midbrain** | 0.055 | 0.526 | 0.152 | 0.322 | 0.079 | 0.358 | -0.261 | 0.422 | -0.094 | 0.855 |
| Abbreviations: PSP = Progressive supranuclear palsy, R = Pearson’s R partial correlation coefficient, Path = neuropathologically confirmed cases, RS = Richardson’s syndrome, DC = diencephalon, SCP = superior cerebellar peduncle.   1. Fisher’s one-tail Z-test p<0.05 vs Subcortical group 2. Fisher’s one-tail Z-test p<0.01 vs Subcortical group 3. Fisher’s one-tail Z-test p<0.001 vs Subcortical group 4. Fisher’s one-tail Z-test p<0.05 vs Cortical group 5. Fisher’s one-tail Z-test p<0.01 vs Cortical group 6. Fisher’s one-tail Z-test p<0.05 vs Cortical group 7. Fisher’s one-tail Z-test p<0.05 vs Richardson’s syndrome group 8. Fisher’s one-tail Z-test p<0.01 vs Richardson’s syndrome group 9. Fisher’s one-tail Z-test p<0.001 vs Richardson’s syndrome group | | | | | | | | | | |

**Supplementary Table 4: Within group Pearson’s r partial correlation coefficients between Progressive Supranuclear Palsy Rating Scale and cortical thickness**

|  | **Cortical thickness vs Progressive Supranuclear Palsy Rating Scale** | | | | | | | | | |
| --- | --- | --- | --- | --- | --- | --- | --- | --- | --- | --- |
| **Region** | **All (n = 80)** | | **Path (n = 28)** | | **RS (n = 62)** | | **Cortical (n = 7)** | | **Subcortical (n = 11)** | |
|  | **R** | **P-value** | **R** | **P-value** | **R** | **P-value** | **R** | **P-value** | **R** | **P-value** |
|  |  |  |  |  |  |  |  |  |  |  |
| **Right Frontal** | 0.159 | 0.136 | 0.158 | 0.502 | 0.198 | 0.085 | 0.235 | 0.795 | -0.455 | 0.066 |
| **Right Parietal** | 0.132 | 0.194 | -0.066 | 0.692 | 0.185 | 0.094 | 0.127 | 0.795 | -0.391 | 0.096 |
| **Right Temporal** | 0.029 | 0.717 | -0.115 | 0.602 | 0.044 | 0.689 | 0.502 | 0.421 | -0.603 | 0.023 |
| **Right Occipital** | 0.132 | 0.194 | 0.142 | 0.514 | 0.179 | 0.094 | 0.183 | 0.795 | -0.427 | 0.071 |
| **Right Cingulate** | 0.247 | 0.020 | 0.477 | 0.003 | 0.337 | 0.002 | 0.384 | 0.584 | -0.517 | 0.050 |
| **Right Insula** | 0.051 | 0.573 | 0.074 | 0.692 | 0.077 | 0.477 | 0.278 | 0.795 | -0.443 | 0.067 |
| **Left Frontal** | 0.087 | 0.370 | 0.004 | 0.975 | 0.119 | 0.251 | 0.202 | 0.795 | -0.562 | 0.032 |
| **Left Parietal** | 0.224 | 0.020 | 0.096 | 0.650 | 0.274 | 0.009 | 0.145 | 0.795 | -0.183 | 0.428 |
| **Left Temporal** | -0.092 | 0.370 | -0.178 | 0.502 | -0.035 | 0.700 | 0.580 | 0.421 | -0.764 | 0.001 |
| **Left Occipital** | 0.069 | 0.464 | 0.170 | 0.502 | 0.150 | 0.148 | -0.003 | 0.993 | -0.485 | 0.052 |
| **Left Cingulate** | 0.221 | 0.020 | 0.282 | 0.221 | 0.280 | 0.009 | 0.470 | 0.421 | -0.369 | 0.109 |
| **Left Insula** | 0.102 | 0.341 | 0.157 | 0.502 | 0.166 | 0.114 | 0.107 | 0.795 | -0.484 | 0.052 |
|  |  |  |  |  |  |  |  |  |  |  |
| Abbreviations: PSP = Progressive supranuclear palsy, R = Pearson’s R partial correlation coefficient, Path = neuropathologically confirmed cases, RS = Richardson’s syndrome, DC = diencephalon, SCP = superior cerebellar peduncle. | | | | | | | | | | |

**Supplementary Table 5: Within group Pearson’s r partial correlation coefficients between Progressive Supranuclear Palsy Rating Scale and cortical area**

|  | **Cortical area vs Progressive Supranuclear Palsy Rating Scale** | | | | | | | | | |
| --- | --- | --- | --- | --- | --- | --- | --- | --- | --- | --- |
| **Region** | **All (n = 80)** | | **Path (n = 28)** | | **RS (n = 62)** | | **Cortical (n = 7)** | | **Subcortical (n = 11)** | |
|  | **R** | **P-value** | **R** | **P-value** | **R** | **P-value** | **R** | **P-value** | **R** | **P-value** |
|  |  |  |  |  |  |  |  |  |  |  |
| **Right Frontal** | -0.314 | <0.001 | -0.580 | <0.001 | -0.287 | 0.005 | -0.469 | 0.211 | -0.493 | 0.061 |
| **Right Parietal** | -0.177 | 0.030 | -0.404 | 0.003 | -0.175 | 0.075 | -0.450 | 0.211 | -0.174 | 0.451 |
| **Right Temporal** | -0.218 | 0.009 | -0.545 | <0.001 | -0.172 | 0.075 | -0.551 | 0.153 | -0.317 | 0.202 |
| **Right Occipital** | -0.260 | 0.002 | -0.547 | <0.001 | -0.227 | 0.023 | -0.371 | 0.317 | -0.394 | 0.132 |
| **Right Cingulate** | -0.316 | <0.001 | -0.498 | <0.001 | -0.319 | 0.004 | -0.734 | 0.026 | -0.519 | 0.061 |
| **Right Insula** | -0.088 | 0.271 | -0.341 | 0.011 | -0.085 | 0.351 | -0.086 | 0.779 | -0.259 | 0.280 |
| **Left Frontal** | -0.351 | <0.001 | -0.653 | <0.001 | -0.291 | 0.005 | -0.756 | 0.026 | -0.526 | 0.061 |
| **Left Parietal** | -0.188 | 0.024 | -0.467 | <0.001 | -0.168 | 0.076 | -0.178 | 0.611 | -0.486 | 0.061 |
| **Left Temporal** | -0.290 | <0.001 | -0.625 | <0.001 | -0.252 | 0.012 | -0.679 | 0.043 | -0.312 | 0.202 |
| **Left Occipital** | -0.235 | 0.005 | -0.444 | 0.001 | -0.195 | 0.053 | -0.476 | 0.211 | -0.382 | 0.132 |
| **Left Cingulate** | -0.273 | 0.001 | -0.504 | <0.001 | -0.275 | 0.006 | -0.322 | 0.379 | -0.435 | 0.098 |
| **Left Insula** | -0.171 | 0.034 | -0.364 | 0.007 | -0.144 | 0.122 | -0.296 | 0.391 | -0.489 | 0.061 |
|  |  |  |  |  |  |  |  |  |  |  |
| Abbreviations: PSP = Progressive supranuclear palsy, R = Pearson’s R partial correlation coefficient, Path = neuropathologically confirmed cases, RS = Richardson’s syndrome, DC = diencephalon, SCP = superior cerebellar peduncle. | | | | | | | | | | |

**Supplementary Table 6: Within group Pearson’s r partial correlation coefficients between ‘temporal stage’ and cortical thickness**

|  | **Cortical thickness vs temporal stage** | | | | | | | | | |
| --- | --- | --- | --- | --- | --- | --- | --- | --- | --- | --- |
| **Region** | **All (n = 80)** | | **Path (n = 28)** | | **RS (n = 62)** | | **Cortical (n = 7)** | | **Subcortical (n = 11)** | |
|  | **R** | **P-value** | **R** | **P-value** | **R** | **P-value** | **R** | **P-value** | **R** | **P-value** |
|  |  |  |  |  |  |  |  |  |  |  |
| **Right Frontal** | 0.020 | 0.913 | -0.047 | 0.703 | 0.123 | 0.394 | -0.705 | 0.001 | -0.418 | 0.021 |
| **Right Parietal** | -0.052 | 0.691 | -0.250 | 0.045 | 0.048 | 0.611 | -0.696 | 0.001 | -0.690 | <0.001 |
| **Right Temporal** | -0.124 | 0.167 | -0.386 | 0.002 | -0.047 | 0.611 | -0.526 | 0.023 | -0.496 | 0.008 |
| **Right Occipital** | -0.067 | 0.665 | -0.097 | 0.513 | 0.049 | 0.611 | -0.700 | 0.001 | -0.533 | 0.004 |
| **Right Cingulate** | 0.048 | 0.691 | 0.046 | 0.703 | 0.117 | 0.394 | -0.373 | 0.105 | -0.393 | 0.028 |
| **Right Insula** | -0.129 | 0.167 | 0.372 | 0.002 | -0.038 | 0.611 | -0.612 | 0.007 | -0.448 | 0.015 |
| **Left Frontal** | -0.041 | 0.702 | -0.257 | 0.045 | 0.040 | 0.611 | -0.765 | <0.001 | -0.442 | 0.015 |
| **Left Parietal** | 0.008 | 0.933 | -0.159 | 0.228 | 0.145 | 0.394 | -0.806 | <0.001 | -0.615 | 0.001 |
| **Left Temporal** | -0.148 | 0.167 | -0.402 | 0.002 | -0.063 | 0.611 | -0.490 | 0.034 | -0.602 | 0.001 |
| **Left Occipital** | -0.063 | 0.665 | -0.043 | 0.703 | 0.064 | 0.611 | -0.858 | <0.001 | -0.607 | 0.001 |
| **Left Cingulate** | -0.138 | 0.167 | -0.270 | 0.041 | -0.078 | 0.611 | -0.541 | 0.021 | -0.320 | 0.074 |
| **Left Insula** | 0.005 | 0.933 | 0.175 | 0.195 | 0.111 | 0.394 | -0.423 | 0.069 | -0.452 | 0.015 |
|  |  |  |  |  |  |  |  |  |  |  |
| Abbreviations: PSP = Progressive supranuclear palsy, R = Pearson’s R partial correlation coefficient, Path = neuropathologically confirmed cases, RS = Richardson’s syndrome, DC = diencephalon, SCP = superior cerebellar peduncle. | | | | | | | | | | |

**Supplementary Table 7: Within group Pearson’s r partial correlation coefficients between ‘temporal stage’ and cortical area**

|  | **Cortical area vs temporal stage** | | | | | | | | | |
| --- | --- | --- | --- | --- | --- | --- | --- | --- | --- | --- |
| **Region** | **All (n = 80)** | | **Path (n = 28)** | | **RS (n = 62)** | | **Cortical (n = 7)** | | **Subcortical (n = 11)** | |
|  | **R** | **P-value** | **R** | **P-value** | **R** | **P-value** | **R** | **P-value** | **R** | **P-value** |
|  |  |  |  |  |  |  |  |  |  |  |
| **Right Frontal** | -0.072 | 0.490 | -0.291 | 0.018 | -0.102 | 0.266 | 0.586 | 0.020 | -0.420 | 0.067 |
| **Right Parietal** | -0.069 | 0.490 | -0.192 | 0.109 | -0.094 | 0.273 | 0.189 | 0.509 | 0.139 | 0.489 |
| **Right Temporal** | -0.040 | 0.584 | -0.215 | 0.084 | -0.078 | 0.316 | 0.588 | 0.020 | -0.296 | 0.150 |
| **Right Occipital** | -0.041 | 0.584 | -0.169 | 0.151 | -0.110 | 0.266 | 0.683 | 0.011 | -0.399 | 0.071 |
| **Right Cingulate** | -0.172 | 0.094 | -0.345 | 0.008 | -0.143 | 0.192 | 0.052 | 0.826 | -0.252 | 0.219 |
| **Right Insula** | -0.002 | 0.976 | -0.222 | 0.084 | 0.032 | 0.665 | 0.089 | 0.772 | -0.296 | 0.150 |
| **Left Frontal** | -0.110 | 0.267 | -0.370 | 0.007 | -0.115 | 0.266 | 0.340 | 0.244 | -0.359 | 0.104 |
| **Left Parietal** | -0.130 | 0.232 | -0.309 | 0.013 | -0.199 | 0.079 | 0.558 | 0.025 | -0.145 | 0.489 |
| **Left Temporal** | -0.123 | 0.232 | -0.313 | 0.013 | -0.155 | 0.192 | 0.392 | 0.175 | -0.301 | 0.150 |
| **Left Occipital** | -0.093 | 0.366 | -0.142 | 0.199 | -0.100 | 0.266 | 0.264 | 0.392 | -0.532 | 0.021 |
| **Left Cingulate** | -0.052 | 0.584 | -0.211 | 0.084 | -0.086 | 0.295 | 0.615 | 0.020 | -0.058 | 0.753 |
| **Left Insula** | 0.048 | 0.584 | -0.159 | 0.164 | 0.136 | 0.192 | -0.238 | 0.417 | -0.424 | 0.067 |
|  |  |  |  |  |  |  |  |  |  |  |
| Abbreviations: PSP = Progressive supranuclear palsy, R = Pearson’s R partial correlation coefficient, Path = neuropathologically confirmed cases, RS = Richardson’s syndrome, DC = diencephalon, SCP = superior cerebellar peduncle. | | | | | | | | | | |

**Supplementary Table 8: Within group Pearson’s r partial correlation coefficients between survival from scan and cortical thickness**

|  | **Cortical thickness vs survival from scan** | | | | | | | | | |
| --- | --- | --- | --- | --- | --- | --- | --- | --- | --- | --- |
| **Region** | **All (n = 80)** | | **Path (n = 28)** | | **RS (n = 62)** | | **Cortical (n = 7)** | | **Subcortical (n = 11)** | |
|  | **R** | **P-value** | **R** | **P-value** | **R** | **P-value** | **R** | **P-value** | **R** | **P-value** |
|  |  |  |  |  |  |  |  |  |  |  |
| **Right Frontal** | 0.123 | 0.127 | 0.084 | 0.543 | -0.084 | 0.497 | 0.779 | 0.001 | 0.677 | <0.001 |
| **Right Parietal** | 0.086 | 0.205 | 0.113 | 0.463 | -0.042 | 0.680 | 0.438 | 0.071 | 0.354 | 0.081 |
| **Right Temporal** | 0.209 | 0.007 | 0.290 | 0.031 | 0.113 | 0.378 | 0.592 | 0.010 | 0.353 | 0.081 |
| **Right Occipital** | 0.120 | 0.127 | -0.008 | 0.946 | 0.078 | 0.497 | 0.604 | 0.010 | 0.219 | 0.304 |
| **Right Cingulate** | -0.077 | 0.233 | -0.088 | 0.543 | -0.129 | 0.322 | 0.152 | 0.522 | -0.081 | 0.659 |
| **Right Insula** | 0.251 | 0.001 | 0.372 | 0.007 | 0.159 | 0.290 | 0.655 | 0.004 | 0.546 | 0.005 |
| **Left Frontal** | 0.126 | 0.127 | 0.215 | 0.153 | -0.028 | 0.702 | 0.690 | 0.002 | 0.544 | 0.005 |
| **Left Parietal** | 0.086 | 0.205 | 0.121 | 0.463 | -0.081 | 0.497 | 0.709 | 0.002 | 0.366 | 0.081 |
| **Left Temporal** | 0.199 | 0.008 | 0.350 | 0.007 | 0.145 | 0.290 | 0.755 | 0.001 | 0.203 | 0.313 |
| **Left Occipital** | 0.106 | 0.173 | -0.040 | 0.783 | 0.032 | 0.702 | 0.568 | 0.014 | 0.194 | 0.313 |
| **Left Cingulate** | 0.099 | 0.193 | 0.144 | 0.390 | 0.072 | 0.497 | 0.229 | 0.398 | 0.291 | 0.160 |
| **Left Insula** | 0.092 | 0.205 | 0.175 | 0.272 | 0.048 | 0.680 | 0.158 | 0.522 | 0.398 | 0.073 |
|  |  |  |  |  |  |  |  |  |  |  |
| Abbreviations: PSP = Progressive supranuclear palsy, R = Pearson’s R partial correlation coefficient, Path = neuropathologically confirmed cases, RS = Richardson’s syndrome, DC = diencephalon, SCP = superior cerebellar peduncle. | | | | | | | | | | |

**Supplementary Table 9: Within group Pearson’s r partial correlation coefficients between survival from scan and cortical area**

|  | **Cortical area vs survival from scan** | | | | | | | | | |
| --- | --- | --- | --- | --- | --- | --- | --- | --- | --- | --- |
| **Region** | **All (n = 80)** | | **Path (n = 28)** | | **RS (n = 62)** | | **Cortical (n = 7)** | | **Subcortical (n = 11)** | |
|  | **R** | **P-value** | **R** | **P-value** | **R** | **P-value** | **R** | **P-value** | **R** | **P-value** |
|  |  |  |  |  |  |  |  |  |  |  |
| **Right Frontal** | 0.016 | 0.971 | 0.030 | 0.790 | 0.111 | 0.200 | -0.716 | 0.002 | 0.025 | 0.994 |
| **Right Parietal** | 0.076 | 0.702 | 0.043 | 0.699 | 0.143 | 0.124 | -0.165 | 0.532 | 0.016 | 0.994 |
| **Right Temporal** | 0.046 | 0.826 | 0.008 | 0.940 | 0.153 | 0.112 | -0.685 | 0.003 | 0.053 | 0.994 |
| **Right Occipital** | -0.010 | 0.971 | -0.059 | 0.594 | 0.109 | 0.200 | -0.647 | 0.006 | 0.033 | 0.994 |
| **Right Cingulate** | 0.066 | 0.702 | 0.137 | 0.218 | 0.106 | 0.200 | -0.207 | 0.458 | 0.179 | 0.994 |
| **Right Insula** | -0.009 | 0.971 | -0.017 | 0.876 | 0.061 | 0.410 | -0.511 | 0.032 | -0.093 | 0.994 |
| **Left Frontal** | 0.079 | 0.702 | 0.105 | 0.344 | 0.155 | 0.112 | -0.530 | 0.028 | 0.110 | 0.994 |
| **Left Parietal** | 0.061 | 0.702 | 0.051 | 0.644 | 0.241 | 0.012 | -0.561 | 0.020 | -0.135 | 0.994 |
| **Left Temporal** | 0.090 | 0.702 | 0.086 | 0.438 | 0.214 | 0.021 | -0.574 | 0.019 | -0.045 | 0.994 |
| **Left Occipital** | 0.064 | 0.702 | 0.021 | 0.851 | 0.112 | 0.200 | -0.116 | 0.625 | 0.001 | 0.994 |
| **Left Cingulate** | -0.001 | 0.986 | -0.050 | 0.654 | 0.070 | 0.410 | -0.761 | 0.001 | 0.162 | 0.994 |
| **Left Insula** | -0.037 | 0.850 | -0.082 | 0.460 | -0.061 | 0.410 | -0.369 | 0.146 | 0.136 | 0.994 |
|  |  |  |  |  |  |  |  |  |  |  |
| Abbreviations: PSP = Progressive supranuclear palsy, R = Pearson’s R partial correlation coefficient, Path = neuropathologically confirmed cases, RS = Richardson’s syndrome, DC = diencephalon, SCP = superior cerebellar peduncle. | | | | | | | | | | |
